# Dental caries and their association with socioeconomic characteristics, oral hygiene practices and eating habits among preschool children in Abu Dhabi, United Arab Emirates — the NOPLAS project

**DOI:** 10.1186/s12903-018-0557-8

**Published:** 2018-06-08

**Authors:** Amal Elamin, Malin Garemo, Andrew Gardner

**Affiliations:** 1 Department of Health Sciences, College of Natural and Health Sciences, Zayed University, P.O. Box 144534, Abu-Dhabi, United Arab Emirates; 2 School of Molecular Sciences, University of Western Australia, Crawley, Perth, WA 6009 Australia

**Keywords:** Dental caries, Oral hygiene practices, High sugar intake, United Arab Emirates, Preschool children, Socio-economic status, Socio cultural factors, Nurseries

## Abstract

**Background:**

Dental caries are a global public health problem and influence the overall health of children. The risk factors for caries include biological, socio-behavioral and environmental factors. This cross-sectional study assessed dental caries and their associations with socioeconomic factors, oral hygiene practices and eating habits among Emirati and non-Emirati children in Abu Dhabi, United Arab Emirates (UAE).

**Methods:**

The stratified sample comprised children aged 18 months to 4 years recruited from 7 nurseries. The World Health Organization (WHO) decayed, missing and filled teeth index (dmft) was used to analyze the dental status of the children. Parents completed a questionnaire regarding demographics, food consumption and oral habits. The study was approved by the Research Ethics Committee at Zayed University, UAE (ZU15_029_F).

**Results:**

A total of 186 children with a mean age of 2.46 years, of which 46.2% were Emirati, participated. Overall, 41% of the children had dental caries. The mean dmft±SD was 1.70 ± 2.81 with a mean ± SD decayed component (dt) of 1.68 ± 2.80 and mean ± SD filled component (ft) of 0.02 ± 0.19. Emirati children showed higher mean dmft, Plaque Index and Significant Carries Index values than non-Emirati children (*P <* 0.000). Low maternal education, rural nursery location, infrequent tooth-brushing, frequent consumption of high-sugar food items and Emirati nationality were factors significantly associated with dental caries.

**Conclusions:**

In this study, 4 out of 10 nursery children were found to have dental caries. Sociodemographic factors, dietary and oral health habits were associated with dental caries. Effective oral health interventions tailored to improve eating habits and the dental screening of children in this age group are imperative to mitigate these concerns.

**Electronic supplementary material:**

The online version of this article (10.1186/s12903-018-0557-8) contains supplementary material, which is available to authorized users.

## Background

Good oral health status at a young age is of the utmost importance for children’s development, overall health and well-being [1]. Epidemiological studies have revealed that dental caries are the most prevalent chronic disease worldwide in the pediatric community and represent a costly burden to health care services [2, 3]. There is ample evidence supporting the fact that the caries status of young, permanent dentition is closely related to the status of the primary dentition, indicating the importance of understanding the risk factors for caries in the early years of life [4, 5].

Dental caries are a multifactorial disease, with many risk factors contributing to their initiation and progression. The risk factors can be categorized as biological, environmental or socio-behavioral [1]. In preschoolers, high consumption of sucrose, sweet drinks, high sugar intake between meals, and frequent snacking have all been associated with dental caries [6, 7]. Additionally, the quality of a child’s oral hygiene practices and the parents’ ability to withhold cariogenic snacks are also factors associated with dental caries [1, 8]. Some studies have found an association between tooth-brushing and lower caries prevalence, although the findings are inconsistent [7, 9, 10]. Moreover, socioeconomic factors such as income, education level and family size impact disease prevalence [11–14]. In developing countries, children from urban areas experience a higher prevalence of dental caries, in contrast to industrialized countries, where the highest caries rates have been observed among deprived social groups and ethnic minorities [2, 15].

The global prevalence of childhood caries varies widely, with the lowest prevalence reported in some Western countries, such as Sweden, Italy and the USA [16]. Conversely, a higher prevalence has been reported in the Middle East, where many countries are still undergoing economic transition and the health care system is still developing [6, 7, 10, 17–20]. Despite the fact that oral health care is free for United Arab Emirates (UAE) nationals, a high prevalence of dental caries (range: 74.1–83%) among 4- to 5-year-olds and a high decayed, missing and filled teeth index (dmft) (range: 3.07–10.9) have been reported in different areas of the UAE [6, 7, 17]. A study conducted in Abu Dhabi in 1998 found a high prevalence of dental caries in children aged 2, 4 and 5 years, but to date, very limited data are available on the dental status of toddlers and preschoolers, indicating a clear knowledge gap [21].

The aim of this cross-sectional study was to assess dental caries and their association with socioeconomic factors, oral hygiene practices and eating habits among Emirati and non-Emirati nursery children aged 18 months to 4 years living in Abu Dhabi, UAE.

## Methods

### Subjects and study design

The data for this cross-sectional study were collected in 2015/2016. The target population was nursery children between 18 months and 4 years of age residing in the capital district of Abu Dhabi. A stratified random sampling design was used where clusters consisted of nurseries stratified geographically across urban, suburban and rural areas. The three strata were proportional to the number of nurseries in each geographical area. Seven nurseries participated, representing the three strata. Access to the parents of the children in the target age group was achieved through face-to-face interaction during pickup and drop offs using bilingual study investigators. Parents were provided with oral and written information about the study prior to them being asked to consent to their child taking part. Data were collected through oral examination and a structured questionnaire. This study is part of a project titled ‘Nutrition, Oral Health, Physical Development, Lifestyle, Anthropometry and Socioeconomic Status’ (NOPLAS).

### Questionnaire

Consenting parents completed a self-administered structured questionnaire in either English or Arabic (see Additional file 1). The questionnaire collected information about socio-economic background (e.g., maternal and paternal education levels, self-rated financial status) and oral hygiene and dental health practices (e.g., details about tooth-brushing, dental visits, past dental history). The questionnaire also asked about eating habits using a 42-item Food Frequency Questionnaire (FFQ) covering all food groups, including 9 high-sugar food items (flavored milk, cakes, biscuits, fruit juices, syrups and cordials, soft drinks, ice cream, chocolate, and sweets). The FFQ contained five response choices: ‘more than 1 time/day’, ‘6–7 times/week’, ‘3–5 times/week’, ‘1–2 times/week’, and ‘fewer than 1 time/week or never’. The mean intake frequency of the nine sugary foods was used to assess the associations between the dental indices and sugary food consumption. Similarly, the mean intake frequency for the other 33 food categories was used as a measure of non-sugary food intake.

### Oral examination

Prior to the field study, the intra-examiner reliability was measured by repeated examinations performed on children to assess the intra-examiner agreement of caries status and dental plaque, using Cohen’s Kappa statistics. Others have suggested an acceptable intra-examiner agreement of κ > 0.61–0.93 for caries [22, 23]. In this study a κ > 0.93 was used as an acceptable intra examiner reliability for caries. The results indicated a complete intra examiner reliability for caries yielding κ = 1.0, while for plaque, κ was calculated as 0.924. The participating children were examined by one trained dentist experienced in working with children under field conditions. The children were examined at the nurseries in the presence of a familiar adult, such as a nurse or teacher, and their nursery friends. To reduce anxiety, the dentist explained to the child what would be done prior to the examination. The World Health Organization (WHO) caries-scoring index for primary dentition, the dmft, was used to describe the dental caries status of each child [24]. Plaque was recorded using the Plaque Index (PI) [25]. Each intra-oral clinical examination was performed with the child seated in a conventional school chair facing a window with sunlight access under standard conventional light with the dentist wearing a headlight. The examination was carried out using a sterilized, disposable set consisting of an illuminated mouth mirror (Denlite, Welch Allyn Ltd., Navan, Co Meath, Ireland) and a blunt ball-ended probe (Diagnostic Probe, Hu-Freidy Dental, Chicago, Illinois, USA) with an end diameter of 0.5 mm. The dentist recorded the findings for each child on a scoring sheet. The assessments were performed only on cooperative and happy children, regardless of parental approval, to ensure the wellbeing of the children.

### Ethical consideration

This study was performed in agreement with the Ministry of Social Affairs, UAE. The study received full ethical approval from the Research Ethics Committee at Zayed University, UAE (ZU15_029_F) and complied with the Declaration of Helsinki Ethical Principles for Medical Research. Permissions and approvals were obtained from nursery management. Prior to participation, the parents were provided with detailed information about the study in Arabic and English. Written consent was obtained for each participant.

### Statistical analysis

The statistical software package SPSS version 24.0 was used for all statistical analyses (IBM Corp., Armonk, NY, UAS 2016). The mean dmft score was used to calculate the Significant Carries Index (SIC) as described by Bratthall [26]. Dental caries, mean dmft and SIC were used to determine the extent of dental caries, and the association of other variables with these indices was evaluated using *t*-tests, Pearson correlations or non-parametric tests, including chi-square tests as appropriate. A *P*-value ≤0.05 was considered statistically significant.

## Results

**Fig. 1:**
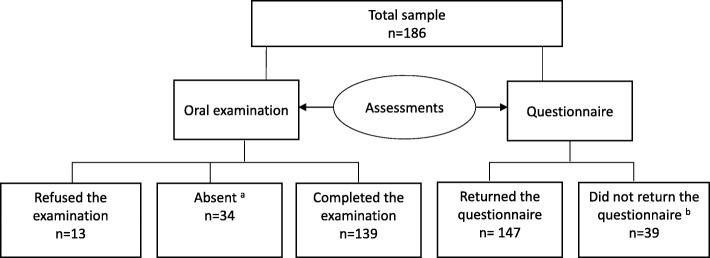
Schematic diagram of the participation in the different assessments among participating nursery children

A total of 186 children (40.9% girls), with a mean age of 2.46 years, participated in the study (Fig. 1). Parents reported their children to be healthy, with no health conditions known to affect oral health status. One-third of the children (34.4%) were enrolled in nurseries located in urban areas, 36.6% in nurseries located in suburban areas and 29.0% in nurseries located in rural areas. Half of the children (54.3%) were > 36 months old, 11.3% were between 18 and 24 months old, and the remaining children (34.4%) were between 25 and 36 months old. The sample had a heterogeneous background, and children were categorized into Emirati children (46.2%) and non-Emirati children (53.8%) based on their nationality, as reported by their parents. The non-Emirati group was comprised of Western, Eastern Mediterranean and Southeast Asian children. The population residing in rural areas is mainly Emirati; thus, the nursery located in this area had mainly Emirati children enrolled, whereas the other locations hosted children of mixed nationalities. There were significant differences in the parents’ education level, as 75.4% of Emirati fathers had a university degree compared to 95.3% of non-Emirati fathers (*P <* 0.01), and the corresponding values for mothers were 63.9% vs. 91.1%, respectively (*P <* 0.01). None of the families considered themselves poor, and 98.6% rated their economic status as middle income, and 1.4% rated themselves as wealthy. A loss analysis revealed that considerably more Emirati families did not return the questionnaire compared to non-Emirati families (34.1% vs. 3.2%, respectively, *P <* 0.001).

Each nursery was visited at least three times for the oral examination, resulting in 74.7% of the children undertaking the dental assessment, with the remaining children refusing the examination or being absent on all three visits (Fig. 1). Overall, 41% of children had dental caries. Decayed teeth (98.7%) contributed the most to the dmft scores. The mean ± SD dmft was 1.70 ± 2.81 with a mean ± SD decayed component (dt) of 1.68 ± 2.80 and a mean ± SD filled component (ft) of 0.02 ± 0.19. There were no missing teeth due to caries (mt). No significant difference was found in the mean dmft between boys and girls, but as shown in Table 1, the Emirati children had considerably more decayed teeth (dt) and higher dmft than the non-Emirati children (*P <* 0.000). Furthermore, the Emirati children had a significantly higher mean dmft when residing in rural areas than those in urban or suburban areas (*P =* 0.03).

**Table 1:** Mean decayed teeth (dt), filled teeth (ft) and dmft scores divided by nationality among nursery children

		Decayed (dt)		Filled (ft)		dmft score^b^	
	Total (n)	*n* (%)	Mean (SD)	*n* (%)	Mean (SD)	*n* (%)	Mean (SD)
All children	139	57 (41.0)	1.70 (2.8)	2 (1.4)	0.02 (0.1)	57 (41.0)	1.68 (2.8)
Emirati children	63^a^	35 (55.6)	2.57 (3.2)	1 (1.6)	0.03 (0.2)	35 (55.6)	2.60 (3.2)
Non-Emirati children	63^a^	16 (25.4)	0.75 (1.8)	0 (0)	0 (0)	16 (25.4)	0.75 (1.8)

*Abbreviations: dmft* decayed, missing and filled teeth index, *dt* decayed teeth*, ft* filled teeth

^a^Thirteen children out of the total of 139 children did not report nationality

^b^No children had missing teeth due to dental caries

**Fig. 2:**
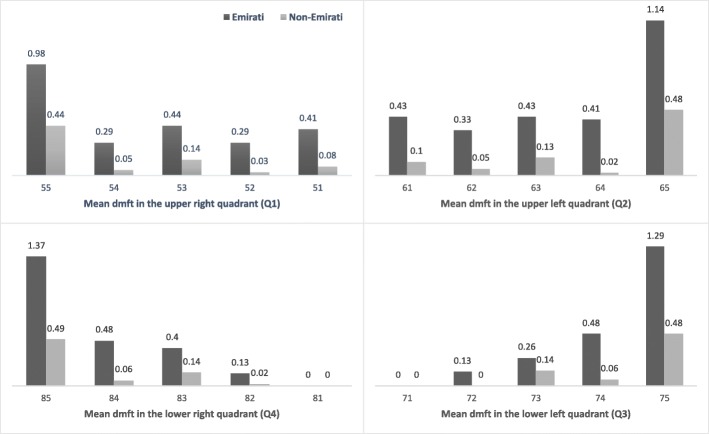
The distribution of the dmft in the four dental quadrants among nursery children

Figure 2 shows the distribution of dmft scores in the four dental quadrants. Dental caries occurred most frequently in the maxillary teeth and in the posterior teeth of both jaws.

**Fig. 3:**
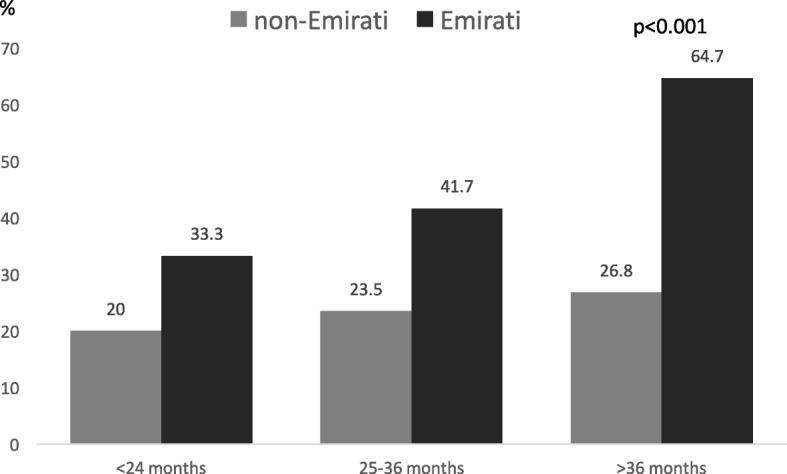
Dental prevalence caries in nursery children divided by age-group and nationality

Figure 3 illustrates the prevalence of dental caries by nationality and age group. Caries were present in children below 24 months of age, and the prevalence increased with age with significant differences between nationalities in children > 36 months old (*P* = 0.001). Table 2 shows the SIC results divided by nationality, gender, nursery location and age group. The PI was considerably higher among Emirati children than non-Emirati children, 1.8 ± 1.0 vs. 0.9 ± 1.0 (*P* < 0.000), with no differences by gender or age.

**Table 2:** Association between Significant Caries Index (SIC) and sample variables among nursery children

	SIC			*X* ^2^	*P*-value
	*n*	%	Mean dmft (SD)		
Nationality				17.29	0.000
Emirati	32	50.8	5.0 (3.0)		
Non-Emirati	10	15.9	4.1 (3.0)		
Gender				2.069	0.15
Boys	32	38.6	4.7 (2.5)		
Girls	15	24.8	5.1 (3.7)		
Nursery				21.677	0.000
Urban	6	12	3.2 (1.2)		
Suburban	19	36.5	4.9 (3.3)		
Rural	22	59.5	5.1 (2.9)		
Age group				3.59	0.166
18–24 months	2	25	3.5 (0.7)		
25–36 months	9	21.4	4.7 (3.6)		
> 36 months	32	37.6	4.9 (3.0)		

*Abbreviations: SIC* Significant Caries Index, *dmft* decayed, missing and filled teeth index, *SD* standard deviation

A majority of the children (75.3%) brushed their teeth at least one time per day, while the remaining children (24.7%) brushed their teeth irregularly or never. There were considerably fewer Emirati children who brushed their teeth daily than non-Emirati children (57.9% vs. 86.5% respectively, *P* < 0.000). Analysis of tooth-brushing habits showed that 52.9% of the children brushed their teeth together with an adult; in 44.3% of these cases, the brushing was done by an adult, and in 2.9% of the cases, the children brushed their teeth by themselves. A vast majority (95.6%) used a regular toothbrush with only 4.6% using an electric toothbrush. More than a quarter of the children (27.9%) had, according to their parents, visited a dentist. Regular checkups were the main reason for the dental visits, with the secondary reason being dental trauma or fissure sealant application. Ten children (6.8%), all Emirati children, reported current dental complaints due to toothache, speech difficulties or habit-related malocclusions. When parents were asked about their perception of their child’s dental health, 91.5% of parents rated the dental health of their child as very good or satisfactory, whereas 7.5% perceived it as dissatisfactory. Emirati parents had a lower perception of their child’s dental health and dental appearance than non-Emirati parents (*P* = 0.009 and *P* = 0.01, respectively). Table 3 shows the associations between dental caries and univariate socioeconomic variables. Maternal education and parents’ perceptions about their child’s dental status were independent variables significantly associated with the mean dmft and SIC values.

**Table 3:** Associations between dental caries and univariate socioeconomic variables among nursery children in Abu Dhabi. *N* = 147

Independent variable	Groups	%	Mean dmft (SD)	*P-*value^a^	Mean SIC (SD)	*P*-value^b^
Father’s education level	High school or below	12.6	2.9 (3.6)	0.062	5.3 (3.3)	0.065
	University degree	87.4	1.2 (2.5)		4.6 (3.2)	
Mother’s education level	High school or below	20.8	3.2 (3.7)	0.000	5.4 (3.40)	0.001
	University degree	79.2	0.9 (2.2)		4.5 (3.0)	
Self-rated financial status	Lower middle income	2.2	2.5 (3.5)		5.0 (−)	0.775
	Middle income	62.6	1.6 (3.0)	0.651	5.4 (0.6)	
	Higher middle income	33.8	1.1 (2.1)		3.9 (2.2)	
	Wealthy	1.4	–		–	
Parents’ perception of their child’s dental status	Very good	41.5	1.0 (1.8)	0.000	3.9 (2.10)	0.000
	Satisfactory	51.0	1.1 (2.3)		4.5 (2.9)	
	Dissatisfactory	6.8	6.9 (4.1)		6.9 (4.1)	
	Very dissatisfactory	0.7	–		0 (0)	

*Abbreviations: dmft* decayed, missing and filled teeth index, SIC significant caries index, *SD* standard deviation

^a^The significance of the dmft scores as measured by the Mann-Whitney U test or Kruskal-Wallis test as appropriate

^b^the significance of SIC scores as measured by Pearson chi-square or Fisher’s exact tests as appropriate

The frequency of consumption of high-sugar food items is shown in Table 4. The intake of sugary foods was positively associated with the dmft (*r* = 0.37, *P* < 0.001). Children who had had caries (dmft> 0) consumed high-sugar food items more frequently than those who were caries free (*P* = 0.003). Children included in the SIC also consumed high-sugar food items more frequently than those who were not (*P* = 0.003).

**Table 4:** Consumption of high-sugar food items based on the FFQ divided by nationality

Food category	Emirati children (*N* = 58)			Non-Emirati children (*N* = 89)			*P*-value
	> 6 times/w (%)	1–5 times/w (%)	< 1 time/w (%)	> 6 times/w (%)	1–5 times/w (%)	< 1 time/w (%)	χ^2^ 2 df
Flavored milk	31.6	42.1	26.3	14.3	21.4	64.3	0.000
Muffins/donuts or similar	25.9	65.5	8.6	9.3	43.0	47.7	0.000
Biscuits/cookies and similar	25.9	58.6	15.5	19.8	54.7	25.6	0.316
Juices	67.3	29.1	3.6	36.8	46.0	17.2	0.001
Syrups/fruit punches/fruit squash	10.5	28.1	61.4	2.4	8.3	89.3	0.000
Soft drinks	3.7	25.9	70.4	1.2	7.1	91.7	0.005
Ice cream	7.1	48.2	44.6	1.1	49.4	49.4	0.159
Chocolates	33.9	51.8	14.3	1.2	62.8	36.0	0.000
Candy/sweets (not chocolates)	23.2	51.8	25.0	4.7	32.6	62.8	0.000

*Abbreviations: FFQ* Food Frequency Questionnaire

## Discussion

Dental health is associated with speech development, eating ability, and overall health in young children [27]. Many caries prediction models have implicated caries in primary dentition as a strong predictor of future caries in permanent dentition [4]. In this stratified sample of children attending nurseries in Abu Dhabi, the prevalence of dental caries was found to be 41%. The univariate analysis revealed that Emirati nationality, low maternal education, rural geographic location of the nursery, and frequent consumption of high-sugar food items were associated with caries in this study population.

The overall mean dmft of 1.7 was lower than that of recent findings by Kowash demonstrating a mean dmft of 10.9 in children below 5 years of age in the Eastern Region of the UAE [6]. While the overall caries status was 41, 64.7% of the Emirati children > 3 years had caries which is on par with regional studies showing that 68–89% of 3- to 5-year-old children were affected by caries [6, 7, 10, 17–20, 28]. The SIC was calculated for one third of the population with the highest caries scores to focus on the group with the most severe caries status [26]. The SIC was found to be almost three times as high as the mean dmft, and while it is always higher, the large difference between the two indices is concerning. A positive association between the dmft index and PI was also found, which is consistent with some studies, whereas others did not find such an association [29, 30].

Culture influences norms of oral health practices, recognition of illness and health care-seeking practices [31]. Although Gulf Cooperation Council countries, including the UAE, have undergone economic transition, it has been suggested that these countries still share some aspects of developing countries such as poor health profiles and low health literacy rates [32]. While caries were found in both nationality groups, all indices (dmft, SIC and PI) in the univariate analyses were significantly higher in Emirati children, as was the consumption of high-sugar food items. Although high sugar intake has been associated with caries in many other studies, it is startling to find a strong association at such a young age, confirming a habit of frequent intake of discretionary calories [33, 34]. One could hypothesize that these findings could be attributed to sociocultural factors, as Emirati families seem to share a practice of frequently including high-sugar food items in their children’s diet. Prediction models exploring specific determinants of dental caries, and potential confounders are essential to fully understand the etiology of dental caries in this population.

Dental status was also related to the level of urbanization and the age of the participants. Children in rural area experienced more caries and had more visible plaque than children in other geographical locations, which is consistent with studies conducted elsewhere [35, 36]. Dental caries were found in children below the age of 2, comparable to studies in Nigeria and Thailand that reported caries in children as young as 12 months of age [37, 38]. In this study, the dmft increased with age indicating the cumulative effect consistent with findings in other studies [39]. The rate of caries in non-Emirati children increased from 20% in children below 2 years of age to 26.8% in children above 3 years of age and the rate of caries in Emirati children almost doubled from 33.3 to 64.7% in the corresponding age groups.

A substantial body of literature has documented an inverse association between socioeconomic status and the of dental caries [14, 29, 40, 41]. Analysis of socioeconomic variables in this study revealed that the mother’s level of education was strongly associated with dental caries*.* However, this inverse association between education level and dental caries was not observed in relation to the father’s education, suggesting the importance of improving health education primarily in mothers [14, 40]. Consistent with previous reports from the region, a toothbrush was the most common brushing aid among children [42, 43]. Tooth-brushing is considered a relatively affordable method of reducing the risk of dental caries mainly via the exposure to fluoride from toothpaste concurrently with mechanical cleansing [44]. In other studies, brushing at least twice daily has been associated with reduced caries occurrence, a finding that could not be confirmed in this study [45]. Despite the fact that oral health services are free for the Emirati population and health insurance is compulsory for non-Emiratis, the utilization of dental services was relatively low in both groups, consistent with other studies conducted in the region [43]. Thus further studies need to focus on understanding how the utilization of dental services can be improved.

This study was conducted in nurseries, i.e., an educational setting under field conditions, which may have strengthened the study design because it is well recognized that hospital-based studies have higher selection bias and subjects are less representative of the general population [46]. Furthermore, the stratified sampling allowed the inclusion of children from areas of differing degrees of urbanization. However, there are some limitation to studies conducted in educational institutes. Unless preschool education is mandatory such studies have no access to subjects who do not attend nurseries. As preschool education is not mandatory in the UAE, the study results cannot be generalized to this segment of the population. As recommended by other researchers, efforts were made to maximize the participation rate by receiving the full support of the nursery administration, the dissemination of electronic and printed invitations, including bilingual investigators and establishing face-to-face contact with parents during regular drop off/pickup times [47]. Repeated visits to the nurseries was also a strategy used to optimize participation. An unexpected challenge related to recruitment was the difficulty accessing parents, unlike what has been reported in other child health studies [47]. In most cultures, parents drop off and pick up their children from nurseries. However, in the UAE, a culture of bringing the children to nurseries by household helpers (e.g., maids and drivers) or by bus was found to be a major limiting factor in accessing large numbers of families, hence the sample size was impacted and a variation in the participation rate was found, as shown in the loss analysis. Accordingly, the sample size can be considered a limitation of this study, suggesting that the results need to be interpreted with caution. The lack of mandatory dental checkups in this age group poses a challenge for accessing large, unbiased groups of children. An alternative strategy could be to recruit children through household visits, which would not necessarily be more efficient for recruitment as it is labor-intensive and costly.

## Conclusions

In conclusion, 4 out of 10 nursery children in this study with a mean age of 2.46 years had dental caries. Lower maternal education, rural nursery location, infrequent tooth-brushing, Emirati nationality and frequent consumption of high-sugar food items were all factors associated with dental caries. The findings that Emirati children consumed significantly more sugar compared to non-Emirati children and had more dental caries may imply the need for targeted interventions. The findings of this study need to be supported by longitudinal, population-based studies. Health education for parents of young children should be considered to improve eating habits. In addition, introducing mandatory dental checkups, starting in toddlers, could be a proactive strategy to screen, prevent and intervene early.

## Additional file

Additional file 1: Questionnaire including demographics, oral health and eating habits. (PDF 97 kb)

## Abbreviations

CI: Confidence interval
dmft: Decayed, missing and filled teeth index
dt: Decayed teeth
FFQ: Food frequency questionnaire
ft.: Filled teeth
mt: Missing teeth
SD: standard deviation
SIC: Significant caries index
UAE: United Arab Emirates
