# Supplementary material for: Dental caries and their association with socioeconomic characteristics, oral hygiene practices and eating habits among preschool children in Abu Dhabi, United Arab Emirates — the NOPLAS project
Source: BMC Oral Health. 2018 Jun 8;18:104. doi: 10.1186/s12903-018-0557-8 (PMC5994070; doi:10.1186/s12903-018-0557-8)
Supplement: Supplementary file 1 — Questionnaire including demographics, oral health and eating habits. (PDF 97 kb) [file 12903_2018_557_MOESM1_ESM.pdf]

**Child's name.......……………………….……..Nursery.......……………………..**
**Interviewer.......………………………..          Date: DD/MM/YY ..................**

---

## A. Contact details

**Contact person.......………………………..………..…………………………………………**

**Relation to Child:  .......………………………..………..…………………………………………**

**Mobile no...........…………Landline no. .......…………………….……........…………**

**E-mail address.......…………………………………………...………………………………**

**Address.......……………………………………………..………………………………………..**

## B. Demographics

1. **Child's date of birth** .......………………………......………………………..

**2. Gender**
   A. Boy
   **B.** Girl

**3.Nationality**:......…………………………..  **Country of origin:** .......……………

**4. How many siblings does the child have?**......…………………..

**5. What is the child's birth order**? …………………………….

**6. Years of residence as a family in UAE (for non-Emiratis)**
   A. 0-5 years
   B. 6-10 years
   C. 11-20 years
   D. More than 20 years

**7. Who does the child live with? (tick all that applies)**
   A. Parent(s)
   B. Uncle/aunt
   C. Grandparent(s)
   D. Siblings
   E. Other(s) Please specify .......………………………..

**8. Marital status of the child's parents:**
   A. Married
   B. Divorced
   C. Widowed
   D. Separated
   E. Other

**9. Mother's occupation**
   A. Housewife

B. Self-employed
C. Part time outside home
D. Full time outside home.

**10. Mother's education:**
A. None
B. Primary school
C. Middle/Secondary/High school or equivalency
D. Bachelor's degree
E. Higher education: Master's degree/Doctorate

**11. Father's occupation....................................................**
A. Stay-home father
B. Self-employed
C. Part time outside home
D. Full time outside home.

**12. Father's education:**
A. None
B. Primary school
C. Middle/Secondary/High school or equivalency
D. Bachelor's degree
E. Higher education: Master's degree/Doctorate

**13. How do you rate yourself financially?**
A. Poor
B. Lower middle income level
C. Middle income level
D. Higher middle income level
E. Wealthy

## C. Eating habits and eating pattern

Think about a typical month in your **child's life** while answering how often your child on average is consuming the following food items. Tick the category that applies to your child's eating habits

| **Food item** | **More than one time/day** | **6-7 times/ week** | **3-5 times/ week** | **1-2 times/ week** | **Less than once per week or never** |
|---|---|---|---|---|---|
| Full fat milk/ yoghurt/Laban | | | | | |
| Low fat milk/ yoghurt/laban | | | | | |
| Flavoured milk (chocolate, strawberry, banana or similar) | | | | | |
| Breastmilk | | | | | |
| Formula milk | | | | | |
| Hard cheese (like cheddar, parmesan) | | | | | |
| Cream cheese/Labnah | | | | | |
| Feta cheese/halloumi/ mozzarella | | | | | |

| | | | | | |
|---|---|---|---|---|---|
| Muffins/Donuts/ Pandesal /chocolate croissants/pancakes/ waffles or similar | | | | | |
| Savoury Croissants(zaata, cheese)/Paratha/ Samosa or similar | | | | | |
| Toast/Lebanese bread/pita bread /bagels/bread/chapatis or similar | | | | | |
| Biquits/cookies/ crackers/ Arabic sweets | | | | | |
| Porridge (oat, barley, wheat, corn or similar) | | | | | |
| Meat (whole pieces) Beef/lamb/camel | | | | | |
| Bacon /Sausages/Hotdogs | | | | | |
| Minced meat/kebabs | | | | | |
| Fatty fish (salmon, tuna, Markel, sardines) | | | | | |
| Lean fish (Hammour, sherry or other white fish) | | | | | |
| Food item | More than one time/day | 6-7 times/ week) | 3-5 times/ week | 1-2 times/ week | Less than once per week or never |
| Lentils/Dried beans/chickpeas/ hummus | | | | | |
| Eggs/omelette/ scrambled eggs | | | | | |
| Yellow/orange/red vegetables (like carrot, pepper, corn, tomatoes,, pumpkin, sweet potatoes ) | | | | | |
| Green vegetables (peas, lettuce, squash, zucchini, spinach) | | | | | |
| White vegetables (onion, cauliflower, potatoes, parsnip or similar) | | | | | |
| Fresh fruits/ fruit salad | | | | | |
| Dried fruits (raisins, apricots, dates, figs) | | | | | |
| Juices (packed or | | | | | |

| | | | | | |
|---|---|---|---|---|---|
| fresh) | | | | | |
| Rice | | | | | |
| Pasta/spaghetti | | | | | |
| Other grains like quinoa, bulgur, barley | | | | | |
| Nutella/ peanut butter | | | | | |
| Chips (crips), Indian spice snack mixes/French fries<br><br>Nuts, almonds | | | | | |
| Butter/margarine for sandwiches | | | | | |
| Mayonnaise | | | | | |
| Mortadella/Smoked turkey/ham/beef (for sandwiches) | | | | | |
| Whip cream/crème fraiche/full fat labnah | | | | | |
| Low fat cream/crème fraiche/low fat labnah | | | | | |
| Food item | More than one time/day | 6-7 times/ week) | 3-5 times/ week | 1-2 times/ week | Less than once per week or never |
| Syrups/fruit punches/fruits squats/TANG | | | | | |
| Soft drinks | | | | | |
| Soft drinks light | | | | | |
| Ice cream | | | | | |
| Chocolates | | | | | |
| Candy /sweets (other than chocolate) | | | | | |

## D. Oral health

**1. How do you rate the health status of your child's mouth and teeth?**
A. Very good
B. Satisfactory
C. Dissatisfactory
D. Very dissatisfactory

**2. Are you satisfied with your child's teeth appearance?**
A. Very satisfied
B. Satisfied
C. Dissatisfied
D. Very dissatisfied

**3. Has your child visited a dentist before?**
A. Yes
B. No

**4. If yes, why did your child visit the dental clinic? (Tick all that applies)**
A. Regular check-ups
B. Toothache
C. Accident/Trauma
D. Swollen gums
E. Loose teeth
F. Prevention treatment to reduce caries
G. Bleeding
H. Others (please specify)…………

**5.What form of treatment did your child receive before in the dental clinic? (Tick all that applies)**
A. Filling
B. Extraction
C. Braces
D. Fissure sealant (preventive treatment)
E. Other (please specify)…………………..

**6. Does you child brush his/her teeth everyday?**
A. Yes
B. No

**7. If yes, how many times a day does your child brush his/her teeth?**
A. Once a day
B. Twice a day
C. Three times a day
D. Other (please specify)

**8. If no, how often does your child brush his/her teeth?**
A. Never
B. Once every two days
C. Once every three days
D. Once a week
E. Irregularly
F. Other(please specify) …………..

**9. Who brushes your child's teeth?**
A. I/Adult brush my child's teeth
B. My child brush her/his own teeth
C. My child brush her/his own teeth with help of an adult

**10. When does the child brush his/her teeth? (Tick all that applies)**

A. My child doesn't brush her/his teeth
B. Morning
C. Afternoon
D. Before going to bed
E. Before meals
F. After meals
**G.** No routine is being followed

**11. What does use child use to brush his/her teeth? (Tick all that applies)**

A. Regular toothbrush
B. Electric toothbrush
C. Dental floss
D. Mouth wash
E. Others(Please specify).................

**12. Does your child currently have any dental complains?**

A. No
B. Yes, **Please specify which of the following (Tick all that applies):**
    A. Pain due to caries
    B. Pain due to trauma/accident
    C. Swollen gums
    D. Loose teeth
    E. Bleeding
    F. Chewing difficulties
    G. Orthodontic problems
    H. Speech problems
    I. Others(please specify)………

**13.How does your child drink liquids? (Tick all that applies)**

A. Bottle
B. Sippy cup
C. Regular cup
D. Others, (Please specify)................................................

**14. Do you use any traditional teething or weaning practices, or any soothing instruments?**

A. Yes, (Please describe)...............................................................................
B. No

## E. General health

Please circle the answer that best applies to you/your child and complete the empty gaps

1. **Birth weight___________**kilogram/pounds **Birth height______________**cm/inches

2. **My child was born in pregnancy week**
    a. < 37 weeks (preterm)
    b. 38-41 weeks (on term)
    c. ≥ 41 weeks (post term)

3. **Was your child breast-fed?**
    a. Yes
    b. No

4. **To my knowledge**
    a. My child has a normal growth
    b. My child has struggled to grow and gain weight

c. My child has increased too much in size

5. **My child is**
   a. Healthy *(skip to Q 8)*
   b. Has a chronic condition, specify what________________________
   c. Has food intolerance/food allergy, specify what________________________________

6. **If your child has any health conditions, has it been diagnosed by a medical doctor**
   a. Yes
   b. No
   c. We know that our child has it thanks to internet/friends/family

7. **My child takes medication on a regular basis**
   a. Yes, specify what and how often____________________________________
   b. No, my child does not take medication unless prescribed by a medical doctor
   c. No, my child is only treated with herbs or other alternative medications if needed

8. **We give our child vitamin and mineral supplementation**
   a. Yes, please specify type __________________________specify since when________________________________
   b. No
